# Volume assessment in critically ill patients: echocardiography, bioreactance and pulse contour thermodilution

**DOI:** 10.1186/cc14256

**Published:** 2015-03-16

**Authors:** S Hutchings, P Hopkins, A Campanile

**Affiliations:** 1 King's College Hospital, London, UK; 2 Papworth Hospital, Cambridge, UK

## Introduction

We performed an evaluation of three devices used for assessment of volume status in critically ill patients in our institution: transthoracic echocardiography (TTE) (CX50; Philips Ultrasound), bio reactance (NICOM; Cheetah Medical) and pulse contour-based thermodilution (PiCCO; Pulsion Medical).

## Methods

Ten mechanically ventilated critically ill patients with PiCCO monitoring *in situ *and a good quality of images on transthoracic view were included. All study measurements were made in triplicate. A single trained cardiologist, blinded to the results from the other monitors, performed the TTE study. Differences among the three methods were assessed for significance using one-way ANOVA, Spearman's coefficient and Bland-Altman analysis. All statistical analyses were performed using Graph-pad Prism 5 and *P *< 0.05 was taken as significant.

## Results

**Figure 1:**
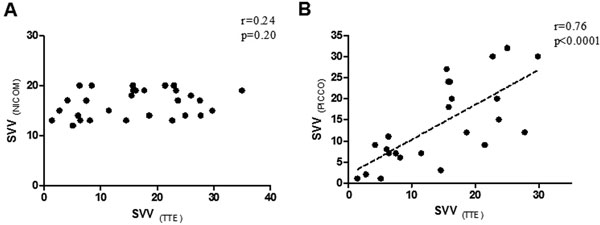


Ninety measurements were obtained. NICOM and TTE-derived stroke volume appeared well matched but PICCO-derived values showed significant variation (*F *= 2.4, *P *= 0.09). There was no correlation between TTE velocity time integer (VTI) and NICOM stroke volume variation (SVV) (*r *= 0.24, *P *= 0.20; Figure 1A) but a good correlation and small bias between TTE-VTI and PiCCO-SVV (*r *= 0.76, *P *< 0.0001; Figure 1B). Applying the following indications for volume expansion (PiCCO and NICOM SVV >15% and TTE VTI variability >15%) we found an agreement in 71% of cases between TTE and PiCCO and in 42% of cases between echocardiography and NICOM.

## Conclusion

Stroke volume produced by bioreactance appeared to be comparable with that measured by echocardiography but not with PiCCO. There was a good agreement between decision-making as regards fluid administration between PiCCO and echocardiography. NICOM appeared unreliable in this setting.

